# Beyond Policy: Strengthening District Level Access to Surgery Is Critical to Achieving Surgical Equity in Universal Health Coverage: Comment on "Improving Access to Surgery Through Surgical Team Mentoring – Policy Lessons From Group Model Building With Local Stakeholders in Malawi"

**DOI:** 10.34172/ijhpm.2023.7594

**Published:** 2023-09-17

**Authors:** Jaymie A. Henry

**Affiliations:** ^1^The Global Alliance for Surgical, Obstetric, Trauma, and Anesthesia Care (G4 Alliance), Chicago, IL, USA; ^2^Division of Cardiothoracic Surgery, Baylor College of Medicine, Houston, TX, USA

**Keywords:** District Level Surgery, UHC, Global Surgery, Essential Surgery, Surgical Equity, Universal Health Coverage

## Abstract

District level access to surgical care has been identified as the rate limiting step to increasing access to the bottom billion and relies on a complex interplay of patient-related and system-based factors that underlie the provision of quality surgical care at point of care. Surgical mentoring via visiting teams, use of current proprietary technologies to enhance communication, establishment of a national surgical coordinator and multi-stakeholder engagement with creative cost-sharing have all demonstrated promising results. Regardless of strategic implementation frameworks, system-based thinking coupled with implementation science with practical solutions will be necessary to inform stakeholders on the best way forward in their respective geographic field of work charting a path towards surgical equity in universal health coverage (UHC).

 The accompanying article by Broekhuizen et al,^1^ is an interesting application of implementation science using a mixed methods approach that codifies the complex web of factors affecting district level surgical care access. The result is a hybrid model on the optimal frequency of surgical mentoring occurrences as well as various recommendations on increasing the effectiveness of the model in low- and middle-income countries. The goal is adoption, ownership, sustainability, and finally, institutionalization of district-level surgical team mentoring within national policy frameworks.

 The issue, strengthening district level access to surgical care, has long been identified as a key strategy to improve equity in surgical service provision^2,3^ and the absence of an accepted proven model has rendered most recommendations, at best, theoretical frameworks. The challenges are understandable, as no ‘one-size-fits-all’ strategy has come to the fore in the last few decades of work, and we are now faced with coming down to discerning nuances and identifying important principles of change. The authors are to be commended, therefore, for their continued focus on this area as well as in the application of scientific rigor to the project. The goal, ultimately, is sustainability and country-level ownership through policy-level actions. The conclusions from the paper can also provide guidance in terms of increasing the efficiency of the mentoring model, providing cost data as well as data on the efficiencies of particular design choices in the rollout of the intervention. Translated properly, this kind of information would be of interest to offices with very strict to almost no budgetary allotment to foster these kinds of activities. Taken in isolation, however, it runs the risk of overshadowing other equally important strategic elements of what it means to build capacity, as training without equipping someone with the means to deliver effective surgical care is akin to teaching someone to fish without supplying them with a boat and a fishing rod. Nevertheless, the overarching recommendations are sound, and perhaps can be expounded and clarified. For example, the authors emphasize the need to “create a focal point with a dedicated national coordinator.” This strategy has been employed in Mongolia^4^ and has served to help increase national surgical capacity by strengthening the *Soum* (the equivalent of districts). Prior to the appointment of a national coordinator, various international organizations were operating independently of each other. In 2006, the Mongolian Ministry of Health launched a national program in partnership with the World Health Organization (WHO) to strengthen emergency and essential surgical care. The coordinator was able to bring together various organizations such as the Swiss Surgical Team, a branch of the International College of Surgeons, the Swiss Government Agency for Development and Cooperation, the Swanson Foundation, and various other organizations, to name a few, to enhance surgical education alongside infrastructure development, resulting in inclusion of 67% of the *aimags *(provinces) and 52.66% of the *soum *(district) hospitals. Post-training and infrastructure development assessments included increase in the fund of knowledge of participants from 47.72% (95% confidence interval 40.7–54.7) to 77.9% (95% confidence interval 70.1–85.7, *P* = .0001) after the training program. There was also a 57.1% increase in the availability of emergency rooms, 59.1% increase in the supply of emergency kits, a 73.64% increase in the recording of emergency care cases, and a 46.66% increase in the provision of facility and instrument usage instructions at the included sites.^4^ It would be useful to clarify if this individual is an employee of the Ministry of Health and is charged with other matters pertaining to surgical capacity building (ie, organizing training programs, camps, interfacing with visiting surgical teams, procurement or supplies, equipment, quality improvement, etc) or is mainly focused on coordinating field visits. This coordinator, as the authors state, can now be the ‘glue’ that binds the districts and the central hospital, creating the hub and spoke model that was mentioned.^6^

 The authors also note field visits of up to four times per year with a corresponding 5% cancellation rate compared with six (25% cancellation rate). This roughly translates to one field visit every three months, with variable patient load available. With the rise of Zoom and WhatsApp technology as ubiquitous and freely available, virtual meetings should be held at least weekly or bi-weekly with conference, morbidity and mortality, and quality improvement meetings. This will allow regular reporting of District Hospital performance as well as provide an opportunity for the mentees to raise concerns and not have to wait for three months before being able to rectify any identified problems. This model is currently being studied in three African countries.^7^ The field visits should be used wisely and have strict parameters, eg, may stack elective complicated cases that could not be referred to the central hospital but need to address urgent or semi-urgent conditions with a specialist available by WhatsApp or Zoom. Moreover, the field visits, scheduled in advance, represents an opportunity for the communities serviced to conduct surgical outreach (eg, door to door campaigns searching for patients living with neglected surgical conditions)^8^ to reduce the backlog and address these conditions before they advance beyond functional repair.

 Several strategies that have been brought up reflect current global recommendations – district level packages of care, hub and spoke referral networks, monitoring and evaluation,^6^ and in the current article, introducing a financing mechanism through which district hospitals are rewarded for more surgical procedures done.^1^ While suggestions on increasing financial incentives for surgical care at the district level may increase the number of surgical care provision, for countries in the low-income bracket who have serious non-surgical competing priorities for resources, this may be not be feasible. The next statements confirm this fact as the authors point out that the source of financing in the districts as mainly donor-driven. Moreover, lessons learned from high-income countries and other low- and middle-income countries have shown that introducing a financing mechanism to reward either hospitals or providers for more surgery (eg, fee-for-service) does not necessarily translate to increased access to quality or safe surgical care, and may in fact, lead to unnecessary procedures designed to maximize income.^9^

 Thus, for countries at this economic stage, creative cost-sharing may fare better than suggesting fee-for-service incentives for providers. As an example, the Neglected Surgical Diseases (NSDs)^8^ project in Meru, Kenya partnered with the Kenya Ministry of Health, the Meru County Government and major non-governmental organizations in addressing the backlog of neglected surgical conditions such as neglected clefts, cataracts, clubfoot, injuries, obstetric fistulas, and hernias and hydroceles. The project involved a multi-stakeholder engagement strategy involving government, academia, non-governmental organizations, and private entities partnering with an established institution such as the College of Surgeons of East, Central, and Southern Africa (COSECSA) in identifying gaps in infrastructure, workforce, and processes required to deliver surgical care safely. In February 2019, 694 community health volunteers undertook door to door screening in five sub-counties of Meru covering 12 189 Meru citizens. An overall prevalence of 30.7% (n = 3748 NSDs) was reported, all with photo identifiers. Prevalence of index NSDs include hernia (7.63%), neglected injury (17.2%), cataract (10.2%), clubfoot (5.56%), fistula (0.6%), cleft (1.3%), and others (8.1%). The elimination of NSDs commenced in June 2019 with a second layer of screening undertaken by the County Health Management Team and Ophthalmic team (n = 1063 clients screened). 232 surgeries were done at the county level while training local ophthalmologic surgeons (n = 214 cataracts, 18 other eye conditions). The follow-up rate post-operatively was 60% with 90% reporting improved vision. The Meru county government equipped eight level four hospitals with a major operating room, onboarded 10 medical doctors and 50 nurses, and has installed 2 surgical residents in the Meru County Level 5 hospital in collaboration with the COSECSA. This model has proved to be an example of utilizing available resources to enhance local surgical capacity building marrying stakeholder engagement with creative cost sharing, building trust with the local government and engendering local ownership of the project.^10^

 In conclusion, accelerated efforts to strengthen district level surgical care whether through local mentoring or other dedicated strategies is critical to achieving surgical equity within universal health coverage (UHC) and in pursuit of several goals and targets set within the WHO’s Thirteenth General Programme of Work 2019–2023 which calls for a global commitment to reach one billion more people with UHC^11^ as well as to renew commitment to the landmark WHO Surgical Resolution 68.15, Strengthening Emergency and Essential Surgical Care as a Component of UHC.^12^ Several proposed strategies are all commendable but will need system-based thinking coupled with implementation science to prove its efficacy according to corresponding environments in which it is applied as well as to institutionalize it within governmental frameworks. We need to move beyond proposing strategies to demonstrating effectiveness and scalability in order to move the needle towards safe, equitable, quality surgical care for all, especially for the bottom billion.

## Ethical issues

 Not applicable.

## Competing interests

 Author declares that she has no competing interests.
